# Variants Identified by Hepatocellular Carcinoma and Chronic Hepatitis B Virus Infection Susceptibility GWAS Associated with Survival in HBV-Related Hepatocellular Carcinoma

**DOI:** 10.1371/journal.pone.0101586

**Published:** 2014-07-02

**Authors:** Cong Li, Xinyu Bi, Ying Huang, Jianjun Zhao, Zhiyu Li, Jianguo Zhou, Meng Zhang, Zhen Huang, Hong Zhao, Jianqiang Cai

**Affiliations:** 1 Department of Abdominal Surgical Oncology, Cancer Institute and Hospital, Chinese Academy of Medical Sciences and Peking Union Medical College, Beijing, China; 2 Department of Etiology and Carcinogenesis, Cancer Institute and Hospital, Chinese Academy of Medical Science and Peking Union Medical College, Beijing, China; MOE Key Laboratory of Environment and Health, School of Public Health, Tongji Medical College, Huazhong University of Science and Technology, China

## Abstract

Recent genome-wide association studies (GWAS) have identified several common susceptibility loci associated with the risk of hepatocellular carcinoma (HCC) or chronic hepatitis B infection (CHB). However, the relationship between these genetic variants and survival of patients with hepatitis B virus (HBV)-related HCC is still unknown. In this study, 22 single nucleotide polymorphisms (SNPs) were genotyped among 330 HBV-related HCC patients using the MassARRAY system from Sequenom. Cox proportional hazards regression was used to examine the effects of genotype on survival time under an additive model with age, sex, smoking status and clinical stage as covariates. We identified four SNPs on 6p21 (rs1419881 T>C, rs7453920 G>A,rs3997872 G>A and rs7768538 T>C), and two SNPs on 8p12 (rs2275959 C>T and rs7821974 C>T) significantly associated with survival time of HBV-related HCC patients. Our results suggest that HCC or CHB susceptibility loci might also affect the prognosis of patients with HBV-related HCC.

## Introduction

Hepatocellular carcinoma (HCC) is the sixth common cancer and the second leading cause of cancer deaths worldwide. The prevalence of HCC varies considerably, with the highest in East, Southeast Asia and Sub-Saharan Africa, and China alone accounts for approximately 50% of all cases [1]. The known etiologies of HCC include chronic infections with hepatitis B or C virus (HBV or HCV), aflatoxin B1 exposure, alcohol consumption, and drug abuse [2]. Among them, chronic infection with HBV is the main etiological factor for developing HCC to majority of HCC cases in China. More than 90% of perinatal infection by vertical transmission results in persistent chronic HBV infection (CHB). In contrast, more than 90% of individuals infected with HBV in adulthood eliminate the viruses spontaneously [3]. Among persistently infected individuals, only 10–30% of individuals with CHB eventually develop liver cirrhosis and HCC. These epidemiology studies indicated that an individual's genetic makeup might play an important role in the HCC carcinogenesis [4].

In the recent years, several genome-wide association studies (GWAS) identified 31 loci in 10 chromosomal regions associated with susceptibility of CHB [5]–[11] or HBV-related HCC [12]–[16]. Evidence has been accumulated to show that some susceptible variants might also be associated with survival time of patients with colorectal cancer, gastric cancer or prostate cancer [17]–[20]. However, relationship between these GWAS-identified susceptibility loci and survival of HCC patients are still unknown. To evaluate the association between CHB or HBV-related HCC susceptibility SNPs and survival in patients with HBV-related HCC, we included a cohort of patients with HBV-related HCC to perform a comprehensive study.

## Materials and Methods

### Study subjects

In this study, 360 patients with HBV-related HCC were recruited from January 1999 to December 2012 at Cancer Hospital, Chinese Academy of Medical Sciences, Beijing, China. Due to lack of some clinicalpathological information, 30 out of the 360 patients (8.3%) were excluded from the study and finally 330 patients were enrolled in the further study. All patients were unrelated ethnic Han Chinese. The diagnosis of HCC was all histopathologically confirmed by at least two local pathologists. We selected the samples for HBV positive but HCV negative according to serology tests and infection history. Serum hepatitis B surface Antigen (HBsAg), hepatitis B surface antibody (anti-HBs), hepatitis B e Antigen (HBeAg), hepatitis B e Antibody (anti-HBe), hepatitis B core antibody (anti-HBc), and antibody to the hepatitis C virus (anti-HCV) were measured by ELISA (Roche, Shanghai, China) according to the manufacturer's instruction. In addition, patients had to meet criteria as follows: a Karnofsky performance score (KPS) of at least 70, a life expectancy of at least three months, and having adequate organ functions. We collected demographic data of each patient such as sex, age, smoking status, and alcohol consumption. Tumor-node-metastasis (TNM) stages were evaluated and classified according to American Joint Commission for Cancer Staging (AJCCS) in 2009, the seventh edition. Individuals who smoked daily for at least one year were defined as smokers, and those who consumed alcohol drinks more than one time per week for over six months were considered drinkers. Written informed consent was obtained from each patient and this study was approved by the Institutional Review Board of Chinese Academy of Medical Sciences Cancer Institute.

### Follow-up study

Overall survival (OS) time of patients was measured from the date of treatment to the date of last follow-up or death. Whether and when a patient had died was obtained from inpatient and outpatient records, patients' relatives, or local Public Security Census Register Office through follow-up telephone calls. The last date of follow-up was 30th December 2013 and no patients were lost. Patients alive on the last follow-up date were considered censored. The median follow-up time was 68 months for this cohort.

### SNP selection and genotype analysis

Genomic DNA were extracted from patients' peripheral blood samples. Blood DNA kit (catalog number: DP31902) was provided by Tiangen Biochemical Technology Co., Ltd. (Beijing, China). The procedure was performed strictly following the manufacturer's instructions. 31 SNPs were reported by six CHB and five HCC susceptibility GWAS in Asian population [5]–[16]. Among them, 22 SNPs with minor allele frequency>0.05 in Chinese Han population were selected based on the genotypes of samples from 1000 Genome project November 2010 ASN (Asian) and genotyped using the MassARRAY system from Sequenom. Genotyping of the other nine SNPs failed because of no appropriate primers for them. Several quality-control measures implemented in genotyping analysis are as follows: duplicated samples were mixed in the plates; persons performing the genotyping assays were not aware of the status of the duplicated samples; both positive and negative (no DNA) control samples were included on every 384­well assay plate; and 20% random samples were genotyped twice by different investigators and all results were completely concordant, with the concordance being 100%.

### Statistical analysis

Kaplan-Meier method was used to estimate survival curves. Log-rank test was adopted to assess the association between SNPs and OS, demographic characteristics, and clinical features. The Cox regression under a log additive genetic model was used to assess the effect of individual SNPs on OS. Hazard ratios (HRs) and 95% confidence intervals (CIs) were calculated with adjustment for age, sex (male or female), KPS (70, 80 or ≥90), smoking status (nonsmoker or smoker), drinking status (nondrinker or drinker), surgery and TNM stage. All data were analyzed using SAS software (version 9.2; SAS Institute Inc., Cary, NC, USA) with two-sided *P*-values (*P*<0.05 were considered significant).

## Results

### Characteristics of the Study Population

The demographic and clinical characteristics of the 330 patients enrolled in this study are shown in Table 1. During the follow-up period, 256 (77.6%) patients died of HCC, with a median survival time (MST) of 18 months. The median age at diagnosis was 54 years (range, 25–88 years). 282 males (85.5%) and 48 females (14.5%) were included. There were 184 smokers (55.8%) and drinkers (68.8%). 65 (19.7%), 126 (38.2%), and 139 (42.2%) patients were with KPS 70, 80, and 90–100, respectively. 90 (27.3%) patients received liver resection, and the other 240 (72.7%) patients received non-surgery treatment including transcatheter arterial chemoembolization (TACE), TACE combined with radiofrequency ablation, or TACE combined with targeted therapy. 100 (30.3%) patients were at stage I, 106 (32.1%) patients were at stage II and 124 (37.6%) patients were at Stage III; corresponding MST were 51, 25, and 10 months, respectively. TNM stage, drinking status, KPS, and surgical treatment showed a significant association with OS in patients with HBV-related HCC (Log-rank *P*<0.05).

**Table 1:** Demographic and Clinical characteristics of 330 patients with HBV-related HCC.

Characteristics	*N* = 330		
	No. (%)	MST (month)	*P* †
Dead	521 (59.6)	18	
Alive	353 (40.4)		
Sex			0.9064
Male	282 (85.5)	18	
Female	48 (14.5)	23	
Age			0.8157
<54 years	164 (49.7)	18	
≥54 years	166 (50.3)	21	
Smoking status			0.0904
Nonsmoker	184 (55.8)	23	
Smoker	146 (44.2)	15	
Drinking status			
Nondrinker	227 (68.8)	24	0.0346
Drinker	103 (31.2)	14	
TNM stage			<0.0001
I	100 (30.3)	51	
II	106 (32.1)	25	
III	124 (37.6)	10	
Surgery			<0.0001
No	240 (72.7)	13	
Yes	90 (27.3)	67	
KPS			0.0049
70	65 (19.7)	11	
80	126 (38.2)	15	
90	139 (42.1)	28	

Abbreviation: No., number of patients; MST, median survival time.

† *P* values for log-rank test.

### Genetic variants associated with OS in patients with HBV-related HCC

**Figure 1:**
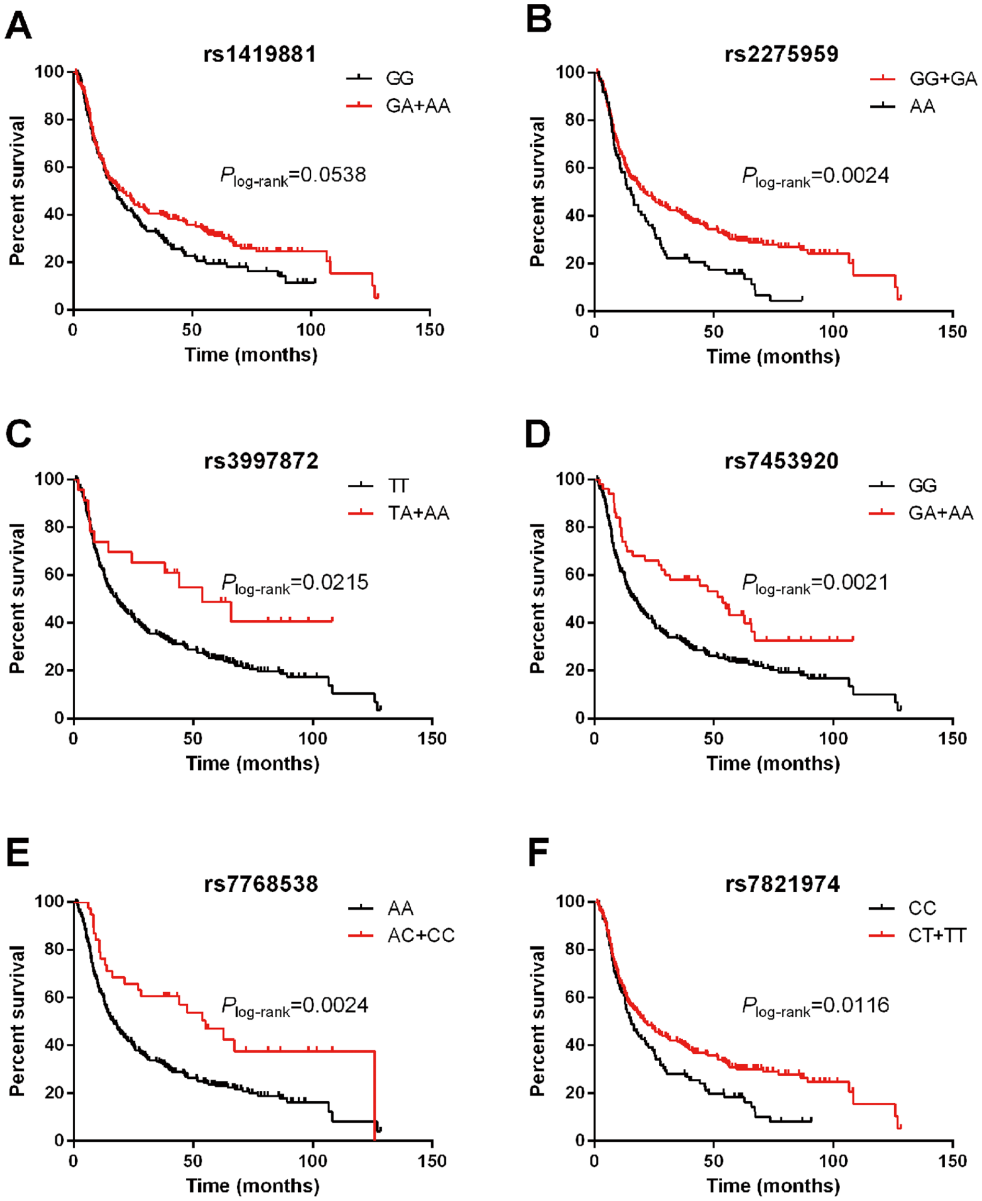
Kaplan-Meier survival curves for patients with HBV-related HCC stratified by genotypes of the six SNPs: A. rs1419881 (did not reach statistical significance for overall survival by log-rank test with *P* = 0.0538 while was significant using the Cox progression model with *P* = 0.00364); B. rs2275959; C. rs3997872; D. rs7453920; E. rs7768538; F. rs7821974.

Among 22 SNPs genotyped, three SNPs (rs1419881 T>C, rs7453920 G>A and rs7768538 T>C) identified by CHB susceptibility GWAS and three SNPs (rs2275959 C>T, rs7821974 C>T and rs3997872 G>A) identified by HCC susceptibility GWAS, were significantly associated with OS in patients with HBV-related HCC in this study using the Cox regression after adjusting for covariates including age, gender, KPS, smoke, alcohol, surgery, and TNM stage (Table 2). (Kaplan-Meier survival curves for patients with HBV-related HCC stratified by genotypes of these six SNPs are shown in Figure 1)

**Table 2:** Associations of 22 candidate SNPs and overall survival in patients with HBV-related HCC.

SNP ID	Chr.	Gene	Minor Allele	MAF	HR (95% CI)†	*P*
rs7768538*	6	HLA-DQB2	C	0.19	0.48 (0.32–0.72)	0.0004
rs7453920*	6	HLA-DQB2	A	0.12	0.52 (0.36–0.75)	0.0004
rs3997872*	6	HLA-DQA1	A	0.06	0.48 (0.25–0.92)	0.0266
rs7821974*	8	LOC101929604	T	0.49	0.81 (0.67–0.98)	0.0335
rs2275959*	8	LOC101929604	A	0.43	1.22 (1.01–1.46)	0.0351
rs1419881*	6	TCF19	A	0.49	0.81 (0.66-0.99)	0.0364
rs3077	6	HLA-DPA1	A	0.38	0.86 (0.70–1.06)	0.1512
rs2647073	6	LOC100507709	C	0.08	1.17 (0.96–1.44)	0.1277
rs6468418	8	LOC101929604	A	0.42	0.87 (0.71–1.06)	0.1737
rs4821116	22	UBE2L3	T	0.36	0.88 (0.71–1.09)	0.2330
rs4678680	3	GLB1	G	0.06	0.79 (0.53–1.18)	0.2505
rs17401966	1	KIF1B	A	0.29	1.12 (0.90–1.40)	0.3003
rs2844619	6	HLA-C	A	0.19	1.07 (0.78–1.48)	0.6798
rs9272218	6	HLA-DQA1	A	0.48	0.94 (0.79–1.11)	0.4435
rs9444730	6	BACH2	G	0.12	0.93 (0.72–1.20)	0.5773
rs9267673	6	C2	T	0.13	1.15 (0.88–1.49)	0.3035
rs7749730	6	BACH2	G	0.12	0.95 (0.73–1.22)	0.6618
rs2724432	11	FDX1	T	0.06	0.90 (0.53–1.52)	0.6876
rs12100561	14	EFCAB11	A	0.38	1.04 (0.86–1.25)	0.7098
rs455804	21	GRIK1	A	0.34	1.04 (0.84–1.28)	0.7346
rs11866328	16	GRIN2A	T	0.18	1.04 (0.82–1.31)	0.7577
rs7574865	2	STAT4	T	0.35	1.00 (0.82–1.23)	0.9916

Abbreviation: MAF, minor allele frequency; HR, hazard ratio; CI, confidence interval.

*The SNPs with *P*<0.05.

† Calculated with multivariate Cox regression under an additive genetic model adjusted for age, sex, smoking status, drinking status, KPS, surgery, and clinical stage.

The rs7768538 A>C locus was the most significant one, with the adjusted HR for death of patients being 0.48 (95% CI, 0.32–0.72; *P* = 0.0004) under the additive model (Table 2). The MST for the rs7768538 AA and AC genotypes was 16 and 54 months, respectively. The adjusted HR for death of patients with the rs7768538 AC genotype was 0.49 (95% CI, 0.31–0.77; *P* = 0.0022), compared with the AA genotype (Table 3). In a dominant model, patients with the rs7768538 AC or CC genotype had significantly longer MST (55 months) than those with the AA genotype, with the adjusted HR being 0.45 (95%CI, 0.29–0.71; *P* = 0.0005) (Table 3).

**Table 3:** HR and MST of patients with HBV-related HCC for the six significant SNPs.

Genotype	No.	Dead/Alive	MST	HR (95% CI)†	*P*
			(months)		
rs7768538					
AA	273	220/53	16	1.00 (Reference)	
AC	36	22/14	54	0.49 (0.31–0.77)	0.0022
CC	3	1/2	na	0.17 (0.02–1.25)	0.0823
AC+CC	39	23/16	55	0.45 (0.29–0.71)	0.0005
rs7453920					
GG	279	225/54	17	1.00 (Reference)	
GA	47	29/18	54	0.55 (0.37–0.82)	0.0030
AA	3	1/2	na	0.16 (0.02–1.19)	0.0740
GA+AA	50	30/20	54	0.51(0.34–0.75)	0.0007
rs3997872					
TT	306	243/63	18	1.00 (Reference)	
TA	22	11/11	66	0.48 (0.25–0.92)	0.0266
AA	0	0/0	na	na	na
TA+AA	22	11/11	66	0.48 (0.25–0.92)	0.0266
rs7821974					
CC	92	81/11	15	1.00 (Reference)	
CT	140	100/40	21	0.64 (0.46–0.88)	0.0059
TT	70	53/17	21	0.69 (0.47–1.00)	0.0525
CT+TT	210	153/57	21	0.66 (0.49–0.88)	0.0046
rs2275959					
GG	91	69/22	21	1.00 (Reference)	
GA	159	114/45	20	1.00 (0.73–1.36)	0.9980
AA	80	73/7	15	1.45 (1.02–2.07)	0.0395
GA+AA	239	187/52	18	1.13 (0.85–1.51)	0.3942
rs1419881					
GG	132	109/23	18	1.00 (Reference)	
GA	151	112/39	19	0.72 (0.55–0.96)	0.0258
AA	45	33/12	24	0.78 (0.51–1.18)	0.2330
GA+AA	196	145/51	21	0.73 (0.56–0.95)	0.0204

Abbreviation: No., number of patients; MST, median survival time; HR, hazard ratio; CI, confidence interval. Because of genotyping failure of some DNA samples, the number of subjects may not add up to the total number.

† Calculated with multivariate Cox regression models adjusted for age, sex, smoking status, drinking status, KPS, surgery and clinical stage.

The rs7453920 G>A, rs3997872 G>A, rs7821974 C>T, and rs1419881 T>C were also significantly associated with survival time in HBV-related HCC patients, with the adjusted HR being 0.52 (95% CI, 0.36–0.75; *P* = 0.0004), 0.48 (95% CI, 0.25–0.92; *P* = 0.0266), 0.81 (95% CI, 0.67–0.98; *P* = 0.0351), and 0.81 (95% CI, 0.66–0.99; *P* = 0.0364), respectively (Table 2). In the dominant model, patients carrying one or two minor alleles would survive significantly longer, compared to those carrying homozygous common genotypes. Patients carrying the rs7453920 G>A, rs3997872 G>A, rs7821974 C>T, and rs1419881 T>C were with the adjusted HR being 0.51 (95%CI,0.34–0.75; P = 0.0007), 0.48 (95%CI, 0.25–0.92; P = 0.0266), 0.66 (95%CI, 0.49–0.88; P = 0.0046), and 0.73 (95%CI, 0.56–0.95; P = 0.0204), respectively (Table 3).

In contrast with the above five loci showing decreased risk of minor alleles on patient's survival, rs2275959 C>T locus was significantly associated with an increased risk of death, with the adjusted HR for death of patients being 1.22 (95% CI, 1.01–1.46; P = 0.0351) under the additive model. In addition, rs2275959 was significantly associated with an increased HBV-related HCC risk in a recessive manner. Patients with TT genotype had significant shorter survival time (MST, 15 months; adjusted HR = 1.50, 95% CI, 1.12–2.01; P = 0.0067) compared to those with the CT or CC genotype.

### Cumulative effects of risk genotypes associated with OS in HBV-related HCC patients

**Figure 2:**
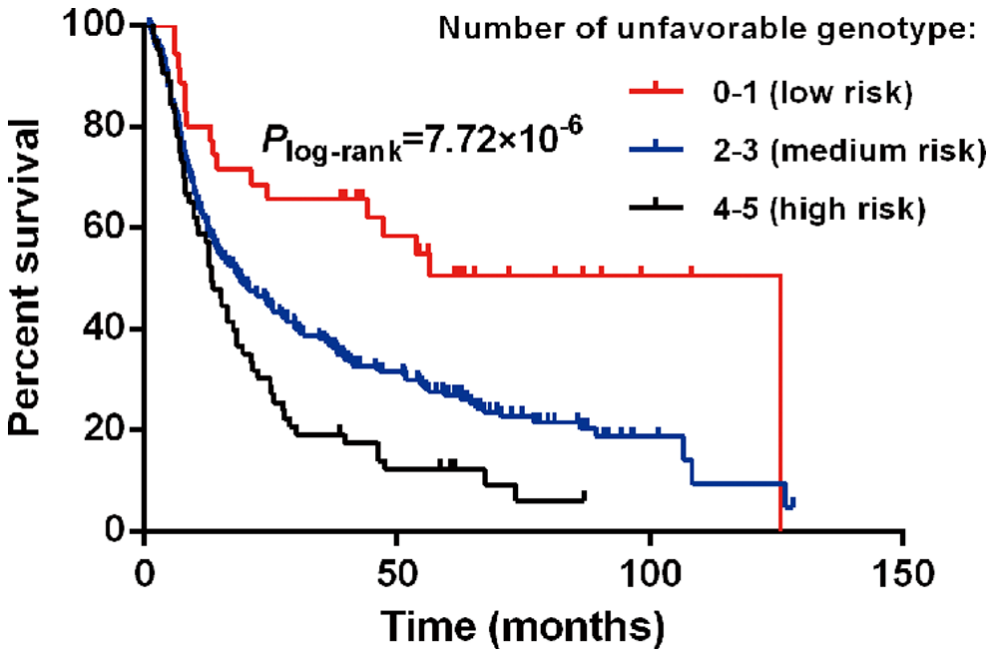
Kaplan–Meier curve of HBV-related HCC patients stratified by the number of unfavorable genotypes.

We further estimated the cumulative effects of risk genotypes associated with OS in HBV-related HCC patients by counting the number of associated genotypes in each subject on the basis of the best-fitting genetic model from single-locus analysis. For rs7453920 and rs7768538 at chromosome 6p21 were in strong linkage disequilibrium (r^2^ = 0.82), we only selected rs7768538, which was more significantly associated with survival in HBV-related HCC patients, for the cumulative analysis. Compared with individuals carrying less than one unfavorable genotype, individuals carrying two or three and four or five unfavorable genotypes had an adjusted HR of 2.14 (95%CI, 1.29–3.581; *P* = 0.0034) and 3.17 (95%CI, 1.77–5.68; *P* = 0.0001), respectively. There was a significant trend of increasing risk of death (*P*_trend_ = 6.23×10^−6^) with increasing number of putative high-risk alleles (Table 4). Kaplan-Meier analysis showed a trend for significantly shorter MST with increasing number of risk alleles for the OS in patients with HBV-related HCC (Log-rank *P* = 7.72×10^−6^; Figure 2).

**Table 4:** Cumulative effect of risk genotypes on survival in HBV-related HCC patients.

Nuamber of risk genotypes	No.	Dead/Alive	MST	HR (95% CI)†	*P*	*P*_log-rank_
			(months)			
0–1 (low risk)	35	17/18	56	1.00 (Reference)		
2–3 (medium risk)	223	173/50	19	2.14 (1.29–3.58)	0.0034	7.72×10^−6^
4–5 (high risk)	72	66/6	13	3.17 (1.77–5.68)	0.0001	
*P*_trend_					6.23×10^−6^	

Abbreviation: No., number of patients; MST, median survival time; HR, hazard ratio; CI, confidence interval.

† Calculated with multivariate Cox regression models adjusted for age, sex, smoking status, drinking status, KPS, surgery and clinical stage.

## Discussion

In this study, we found that GWAS-identified CHB and HCC susceptibility SNPs (rs2275959 and rs37821974 on 8p12; rs1419881, rs3997872, rs7453920, and rs7768538 on 6p21) may individually or jointly affect clinical outcomes for HBV-related HCC patients. To the best of our knowledge, this is the first study to report the association of CHB and HCC susceptibility loci with survival in patients with HBV-related HCC.

rs2275959 C>T and rs7821974 C>T are two susceptibility loci on 8p12 for HBV-related HCC identified by a GWAS in Southern Chinese population. Region 8p was one of the most frequently deleted regions in HCCs, and the loss of heterozygosity in 8p was associated with metastasis of HCC [21]. Moreover, a liver cancer suppressor gene named DLC-1, which induces apoptosis and exerts inhibitory effects on the cell proliferation of HCC cells [22],[23], is located in a site of ∼24 Mb upstream of the two SNPs (rs2275959 and rs37821974) on 8p12. Therefore, we suppose that risk-associated 8p12 SNPs might have an interacting effect on the DLC1 locus. Our data show that the variant genotypes of rs2275959 and rs37821974 respectively increase and decrease the risk of death in HBV-related HCC patients, indicating that the two SNPs might play different roles on the interaction with DLC-1. Additionally, Chan et al found a 2.3-kb expressed sequence tag (EST) in the region 8p12 using in-silico data mining, sequencing analysis and in-vitro protein translation study, which suggested that the transcript might function as a long non-coding RNA (lncRNA) [14]. Accumulating studies have demonstrated that lncRNAs might be dysregulated in HCC and closely related with carcinogenesis, metastasis, or prognosis [24]–[26]. However, the biological and molecular mechanisms of lncRNAs are not yet fully understood. The relationship between 8p12 SNPs and lncRNA on the prognosis in HBV-related HCC patients is unknown and further studies are required.

rs3997872, which was located in a site of ∼23 kb downstream of HLA-DRB1 in the major histocompatibility complex (MHC) class II locus, was associated with the decreased risk of death in patients with HBV-related HCC. MHC class II molecules present antigen to CD4^+^ T cells. Clifford et al discovered that rs3997872 and other two SNPs might be associated with altered MHC class II proteins that result in an ineffective T-cell response and be involved in antigen processing and presentation pathway [12]. Immune response has been proven to play an important role on development and clinical outcomes of HCC [27]–[29]. Furthermore, HLA-DR expression was one of the independent risk factors for early intrahepatic recurrence of HCC, which was strongly in correlation with poor prognosis of HCC [30]. These previous findings enhance the biological plausibility that genetic polymorphism in MHC class II region may play an important role on survival in HCC patients.

Due to only a subset of CHB patients develop HCC, it is of great interest to identify whether genetic factors of CHB susceptibility have an effect on the prognosis of HBV-related HCC patients. We identified three SNPs on 6p21, including rs7453920 (HLA-DQB2), rs7768538 (HLA-DQB2), and rs1419881 (TCF19), whose variant allele frequencies significantly associated with decreased death risk in such patients. rs7453920 and rs7768538 are situated in HLA-DQ genes, which are highly polymorphic, especially in exon 2 which encodes antigen-binding sites. Recent studies have showed that HLA-DQ molecules might be critical for the HBV clearance and play an important role in the development of CHB [9],[15]. HBV-DNA has been confirmed as the most important predictor of disease progression and HCC development in CHB whereas the benefit of HBV clearance for HCC patients' survival time is still far from completely understood [31]. Our findings provided a novel insight that genetic variants in HLA-DQ locus might influence the long-term outcomes in patients with HBV-related HCC through impact of HBV clearance.

rs1419881 on 6p21.3 is located in the 3' untranslated region of transcription factor 19 (TCF19). TCF19 was suggested as a late growth-regulated gene related to the risk of type 1 diabetes and psoriasis vulgaris, playing an important role in the transcription of genes required for the later stages of cell cycle progression [32]–[35]. However, whether TCF19 correlated to survival of HBV-related HCC remains uncertain, and further functional analysis is warranted to fully clarify the molecular mechanism whereby these variations confer survival of HBV-related HCC.

Since the process in clearance of HBV, development of CHB, and progression of HCC are complex, it is unlikely that any single variation would have a dramatic effect on clinical outcomes of HBV-related HCC. However, a combined influence of several SNPs may exert synergistic protection against the development or progression of HCC. Our results support the cumulative effects of risk-associated genotypes of SNPs in modulating survival time of HBV-related HCC, suggesting that screening of these SNPs may provide valuable information in clinical practice to predict different death risk for such patients.

In summary, this is the first study exploring the association of GWAS-identified HCC and CHB susceptibility SNPs with clinical outcomes of patients with HBV-related HCC. GWAS-identified SNPs, including rs2275959, rs37821974, rs1419881, rs3997872, rs7453920, and rs7768538, are significantly associated with survival time of HBV-related HCC. The GWASs from which these SNPs derived are all based on Asian population, making this study more reasonable. Results from cumulative analysis show a gene-dosage effect in these SNPs. Future independent validations in larger population are necessary before these findings translated into clinical practice and beneficial to prognosis in such patients.

## Supporting Information

Table S1 Primers for genotyping of candidate SNPs.
(XLS)
